# Tumour Cannabinoid CB_1_ Receptor and Phosphorylated Epidermal Growth Factor Receptor Expression Are Additive Prognostic Markers for Prostate Cancer

**DOI:** 10.1371/journal.pone.0015205

**Published:** 2010-12-23

**Authors:** Christopher J. Fowler, Peter Hammarsten, Anders Bergh

**Affiliations:** 1 Department of Pharmacology and Clinical Neuroscience, Pharmacology, Umeå University, Umeå, Sweden; 2 Department of Medical Biosciences, Pathology, Umeå University, Umeå, Sweden; University of California Los Angeles and Cedars-Sinai Medical Center, United States of America

## Abstract

**Background:**

In cultured prostate cancer cells, down-regulation of epidermal growth factor receptor (EGFR) has been implicated in mediating the antiproliferative effect of the endogenous cannabinoid (CB) ligand anandamide. Using a well-characterised cohort of prostate cancer patients, we have previously reported that expression levels of phosphorylated EGFR (pEGFR-IR) and CB_1_ receptor (CB_1_IR) in tumour tissue at diagnosis are markers of disease-specific survival, but it is not known whether the two markers interact in terms of their influence on disease severity at diagnosis and disease outcome.

**Methodology/Principal Findings:**

Data from a cohort of 419 patients who were diagnosed with prostate cancer at transurethral resection for voiding difficulties was used. Scores for both tumour CB_1_IR and pEGFR-IR were available in the database. Of these, 235 had been followed by expectancy until the appearance of metastases. For patients scored for both parameters, Cox proportional-hazards regression analyses using optimal cut-off scores indicated that the two measures provided additional diagnostic information not only to each other, but to that provided by the tumour stage and the Gleason score. When the cases were divided into subgroups on the basis of these cut-off scores, the patients with both CB_1_IR and pEGFR-IR scores above their cut-off had a poorer disease-specific survival and showed a more severe pathology at diagnosis than patients with high pEGFR-IR scores but with CB_1_IR scores below the cut-off.

**Conclusions/Significance:**

These data indicate that a high tumour CB_1_ receptor expression at diagnosis augments the deleterious effects of a high pEGFR expression upon disease-specific survival.

## Introduction

The endogenous cannabinoid (CB) system in the body consists of two G-protein coupled CB receptors, CB_1_ and CB_2_, their endogenous ligands anandamide (arachidonoylethanolamide) and 2-arachidonoylglycerol, and their synthetic and degradative enzymes. Whilst much is known about the role of the endocannabinoid system in the brain and its potential for the design of novel analgesic drugs, among others, evidence is emerging that it may play an important role in the pathogenesis and possibly treatment of cancer [1]-[3]. In prostate cancer cells, for example, activation of CB receptors usually [4]–[8] but not invariably [9] leads to inhibition of basal and/or stimulated cell proliferation. An increase in the local endocannabinoid concentration (by blockade of their metabolism) results in a reduced invasivity of the cells *in vitro*, whilst reduction of 2-arachidonoylglycerol synthesis, blockade of CB_1_ receptors, or an increased expression of the anandamide metabolising enzyme fatty acid amide hydrolase produces the reverse pattern [10], [11]. Taken together, these studies suggest that in the prostate, there is a local protective endocannabinoid tonus. Consistent with this hypothesis, expression of epithelial fatty acid amide hydrolase, the enzyme responsible for the metabolism of anandamide, is higher in prostate cancer tissue than in normal prostate tissue, and transfection of androgen-insensitive PC3 prostate cancer cells increases their invasivity *in vitro* [12].

The epidermal growth factor receptor (EGFR) is a cell surface receptor tyrosine kinase responsive to a number of growth factors, including epidermal growth factor, transforming growth factor α and amphiregulin. Phosphorylation of EGFRs leads to activation of a number of different intracelluar signalling pathways, in turn resulting in cell growth and survival [13]. Disturbed EGFR signalling, due for example to the overexpression of EGFR, is involved in the pathogenesis of several cancer types, and antibodies directed towards the extracellular domain of EGFR have been developed for the treatment of cancers such as advanced colorectal cancer [13], [14]. In the prostate, higher levels of epithelial EGFR immunoreactivity (EGFR-IR) were seen in prostatic adenocarcinoma than in normal tissue [15], and patients with a tumour tissue pEGFR-IR score in the top 66% bracket showed a poorer disease-specific survival than those cases with a pEGFR-IR score in the bottom 34% [16].

Very little is known about the link between cannabinoid and EGFR signalling in cancer, and nothing is known in this respect in human tumour tissue. To our knowledge, the only study undertaken in prostate cancer cells is that reported by Mimeault *et al.* [5]. These authors found that anandamide inhibited EGF-stimulated cell proliferation of LNCaP, DU145 and PC3 prostate cancer cells in a manner blocked by pertussis toxin (implicating a G_i_-coupled receptor) and by the CB_1_ receptor inverse agonist rimonabant, but not by the CB_2_ receptor inverse agonist SR144528. Furthermore, in all three cell lines, anandamide treatment reduced the expression of EGFR, again in a manner blocked by rimonabant [5]. Given the local protective role of endocannabinoids in the prostate (see above), this study raises the possibility that differences in the relative expression of CB_1_ receptors and pEGFR in the tumour tissue may affect the pathogenesis and outcome of the disease.

At Umeå university, we have access to a large series of formalin-fixed, paraffin-embedded samples of prostate tumour and non-malignant tissue that were obtained at diagnosis from patients undergoing transurethral resection for micturation difficulties. The patients were followed for up to 23 years, in many cases by active expectancy (watchful waiting) until the appearance of metastases, this being the treatment paradigm at the time [17]. This material allows the study not only of the association of biochemical parameters with disease severity at diagnosis, but also of their association (and potential prognostic usefulness) with disease-specific survival. These samples were used for the tumour epithelial pEGFR study described above [16], but have also been used by us to investigate CB_1_ receptor immunoreactivity (CB_1_IR) in prostate cancer, where a high tumour expression level was associated with a poorer disease-specific survival [18]. Thus, both parameters were measured in the same patient set, and in a simple correlation matrix, we noted that tumour epithelial pEGFR and CB_1_IR were significantly correlated [19]. However, it is not known whether the two parameters provide additive or alternatively overlapping prognostic information, and whether cases with different levels of CB_1_IR for a given pEGFR-IR show different degrees of disease severity upon diagnosis. In consequence, we have reanalysed the raw data from [16] and [18] to answer these questions.

## Methods

### Ethics Statement

The research ethical committee at Umeå university hospital (Regional Ethical Review Board in Umeå, Sweden) approved of the studies and waived the need for informed consent.

### Patient material and immunochemistry

The tumour epithelial CB_1_IR and pEGFR-IR scores used in the present study were taken from our database, the original data for CB_1_IR and pEGFR-IR having been published previously [16], [18]. Readers are referred to those papers for a detailed description of the samples and immunohistochemical techniques used. The tissue material was collected at the Regional Hospital, Västerås, Sweden, between 1975 and 1991, and the patients were followed until 2003. Tissue microarrays were constructed and in general between 1 and 8 cores (usually 5) (tumour tissue) and 1–4 cores (non-malignant tissue) could be scored for the parameter in question. CB_1_IR was scored on the basis of intensity (0 =  absent up to 3 =  high intensity) × distribution, giving a range of 0–3. The median value for the cores scored for a given patient were then entered into the database. pEGFR was also scored on the basis of intensity and distribution, but in this case the range was 0–5. In both cases, the scores were provided by investigators who were blind to the patient data.

### Statistical evaluations

Receiver operating characteristic (ROC) curves, Kaplan-Meier survival analyses, correlation coefficients and χ^2^ tests were undertaken using the statistical package built into the GraphPad Prism 5 computer programme for the Macintosh (GraphPad Software Inc., San Diego, CA, USA). Cox proportional-hazards regression analyses, were conducted using SPSS software (SPSS Inc., Chicago, IL, USA). For survival analyses, an event was defined as death due to prostate cancer (shown in the figures as “†_p_”). Death from other causes was censored, as were the cases where the patient was still alive at the date of last follow-up. Cases (n = 3) where the disease outcome was unknown were excluded from the survival analyses. The duration of event-free survival is defined as the time from diagnosis until either the date of prostate cancer death, death of other causes, or if no death occurred, until the date of last follow-up.

## Results

### Correlation of CB_1_IR and pEGFR-IR in prostate cancer samples

**Figure 1:**
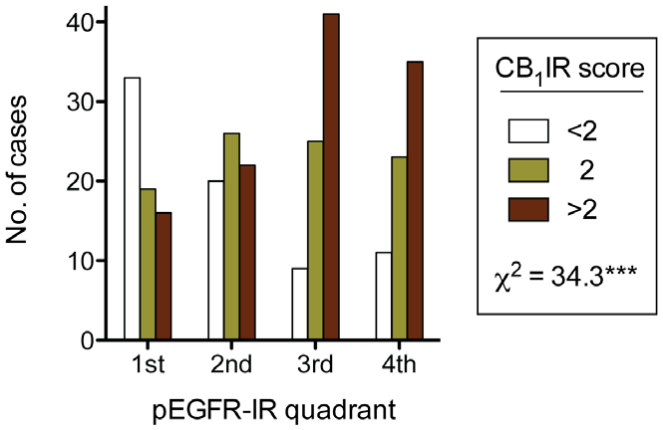
Distribution of CB_1_IR scores for different pEGFR scores in prostate tumour tissue. The pEGFR scores were divided into quadrants (1st, pEGFR <2.6, n = 68; 2nd, 2.6–3.29, n = 68; 3rd 3.3–3.7, n = 75; 4th, >3.7, n = 69) and the CB_1_IR score at each quadrant shown. The number of cases with CB_1_IR scores <2, 2 [the median value] and >2 were 73, 93 and 114, respectively. ***p<0.001, χ^2^ test.

Out of a total of 419 cases in the database, 372 were scored for tumour CB_1_IR [18] and 300 for tumour pEGFR-IR [16]. The CB_1_IR scores ranged from 0–3 units (median 2) and the pEGFR-IR scores from 0-5 units (median 3.3). The significant correlation between tumour CB_1_IR and the tumour pEGFR-IR in the 280 cases where both parameters were scored (Spearman's ρ = 0.316, p<0.001) found in our initial investigation, and which sparked the present analysis [19], can simply be visualised by dividing the pEGFR-IR scores into quadrants and comparing the CB_1_-IR at each score (Fig. 1). Non-malignant CB_1_IR was also correlated with the non-malignant pEGFR-IR (ρ = 0.183, p<0.01 for luminal pEGFR and ρ = 0.159, p<0.01 for basal pEGFR, n = 264).

### Disease-specific survival: influence of CB_1_IR and pEGFR-IR

Of the patients recorded in the database, 307 had been followed with expectancy until the development of metastases rather than being given a curative treatment, this being the standard therapeutic approach at the time. These patients provide a useful resource with which to assess the prognostic value of biomarkers. Of these, 269, 253 and 235 were scored for tumour CB_1_IR, tumour pEGFR-IR and both parameters, respectively, cases where patient outcome was not known having been excluded.

**Figure 2:**
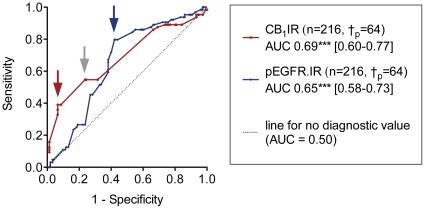
Receiver operating characteristic (ROC) curves for tumour CB_1_IR and pEGFR-IR. For each curve, the number of cases used in the analysis (which used a 15 year limit) together with the mean AUC value is shown. Values in square brackets are the 95% confidence intervals for the AUCs. †_p_ refers to the number of cases who died as a result of the prostate cancer. The arrows show the part of the curve corresponding to the optimal cut-off values: red  =  Youden index for CB_1_IR, grey  =  least squares method for CB_1_IR, blue  =  both Youden and least squares method for pEGFR-IR (for details, see Results section of this paper).

A standard way of assessing the prognostic utility of a biomarker is to use a receiver operating characteristic (ROC) analysis. ROC analyses were originally developed to aid the interpretation of radar signals, and plot the number of true negatives (termed “1-specificity”) vs. the number of true positives (termed “sensitivity”) for all the possible cut-off values for the data set. The area under the curve (AUC) of the resulting graph will be somewhere between 0.5 (no prognostic value) and 1.0 (a perfect test) [20]–[22]. The ROC curves, with a 15 year cut-off, for tumour CB_1_IR and pEGFR-IR values (only cases scored for both CB_1_IR and pEGFR-IR were included in the analyses) are shown in Fig. 2. As expected from our original data [16], [18], the AUC for both CB_1_IR and pEGFR were significantly greater than 0.5.

Having obtained a ROC curve with an area under the curve significantly greater than 0.5, a cut-off can then be chosen for which to investigate the influence of the biomarker upon disease-specific survival. The choice of cut-off is a trade-off between the cost (in terms of discomfort to the patient) of treating false positives (the number of which increase as the cut-off value decreases) with that of missing false negatives (the number of which increase as the cut-off value increases) [20]–[22]. In our initial studies, we used cut-off values of < and ≥2 (for CB_1_IR) and < and ≥2.78 (for pEGFR) were used, since these values were the lowest scores giving a specificity >0.5 [16], [18]. However, when investigating the interaction between two potential prognostic markers, the most appropriate cut-off value is the optimal value for each parameter in question, i.e. the point nearest the top left of the graph shown in Fig. 2. The two methods most commonly used to quantify the optimal cutoff are the least squares method (minimum value of (1-sensitivity)^2^ + (1-specificity)^2^) and the Youden index (the maximum score of (specificity + sensitivity -1)). The two methods give identical cut-offs in some cases, but not in others [22]. In the case of pEGFR, both methods gave an optimal cut-off of >3.172 (shown as the blue arrow in Fig. 2), i.e. division of the samples into two groups with scores <3.2 and ≥3.2 In the case of CB_1_IR, the Youden index (red arrow in Fig. 2) was >2.275, i.e. division of the samples into two groups with scores <2.3 and ≥2.3, whilst for the least squares method, the cut-off was slightly lower (>2.088, grey arrow in Fig. 2). It has been argued that the Youden index is more appropriate in a clinical setting than the least squares method [22] and we have in consequence used the Youden index here for our further analyses. Division of the samples into test and validation sets with which to test the prognostic usefulness of the markers gave the same Youden index values (see supporting Fig. S1 and legend for the data with CB_1_IR).

In survival analysis, Cox proportional-hazards regressions are used to assess the contribution of different prognostic markers upon the measured end-point (here death due to prostate cancer) without making assumptions about the shape of the survival curve. Here, we used Cox proportional-hazards regression analyses to establish whether or not the tumour CB_1_IR provided additional prognostic information to that supplied by pEGFR-IR (Table 1). Using the optimal cut-off values determined in the ROC analyses, we found this to be the case, and the two parameters also gave additional prognostic information to that supplied by the tumour stage and the Gleason score.

**Table 1:** Cox proportional-hazards regression analyses for tumour epithelial CB_1_IR and pEGFR-IR scores.

Variable		n	Exp(B)	95% CI	Variable		N	Exp(B)	95% CI
*Univariate analyses*					*Bivariate analysis*				
CB_1_IR	<2.3	225			CB_1_IR	<2.3	199		
	≥2.3	44	4.40***	2.69–7.21		≥2.3	36	3.75***	2.24–6.29
					pEGFR	<3.2	108		
pEGFR	<3.2	108				≥3.2	127	3.01***	1.61–5.61
	≥3.2	127	3.85***	2.09–7.08					
					*Multivariate analysis*				
T^a^	T1a–T1b	162			CB_1_IR	<2.3	198		
	T2	71	3.66***	2.03–6.58		≥2.3	36	2.64**	1.53–4.58
	T3	31	11.2***	5.86–21.3	pEGFR	<3.2	108		
	T4	3	10.7*	1.40–81.5		≥3.2	126	2.10*	1.12–3.94
					T^a^	T1a–T1b	142		
GS^b^	4–5	78				T2	60	1.38^NS^	0.70–2.73
	6–7	132	22.7**	3.1–166		T3	29	2.86**	1.33–6.17
	8–10	59	139***	18.9–1020		T4	3	4.87^NS^	0.62–38.2
					GS^b^	4–5	48		
						6–7	130	13.4*	1.80–99.2
						8–10	56	46.6***	6.04–360

Analyses were carried out using data (reported in [16] and [18]) from patients who were followed by expectancy until the appearance of metastases.

a Tumour stage,

b Gleason score (in both cases, as well as for pEGFR, the sample sizes used in the univariate analyses are for those which were scored for CB_1_IR). Exp(B) refers to the increase in the odds as a result of an increase in the “unit” of the predictive variable under study.

***p<0.001,

**p<0.01,

*p<0.05,

NS p>0.1.

**Figure 3:**
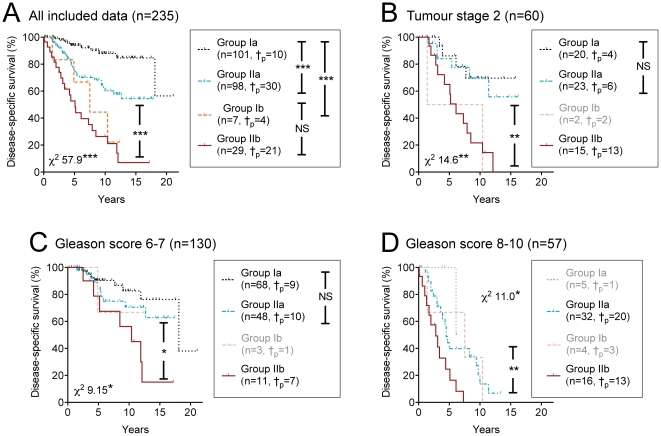
The influence of pEGFR-IR and CB_1_IR scores upon disease-specific survival of patients with prostate cancer. Panel A, all cases; B, cases with tumour stage 2; C, cases with Gleason scores of 6 or 7; D, cases with Gleason scores of 8–10. Scores are shown as CB_1_IR/pEGFR Group Ia (pEGFR-IR <3.2, CB_1_IR <2.3), Group IIa (pEGFR ≥3.2, CB_1_IR <2.3), Group Ib (pEGFR-IR <3.2, CB_1_IR ≥2.3) and Group IIb (pEGFR ≥3.2, CB_1_IR ≥2.3). †_p_ refers to the number of cases who died as a result of the prostate cancer. The χ^2^ values shown in the panels are from the log rank (Cox-Mantel) test. Individual comparisons between two groups were also undertaken. The symbols given between the capped line in the figure itself indicate a comparison (log rank (Cox-Mantel) test) for Group IIa vs. Group IIb (i.e. between the blue and dark red survival curves), whilst the symbols between the capped lines in the legends indicate significance levels for the comparisons shown. When the group size was ≤5, the curves are shown in a lighter colour, and whilst the data were included in the total χ^2^ statistic (bottom left in Panels A–C, top right in Panel D), individual comparisoms were not undertaken. ***p<0.001, **p<0.01, *p<0.05, ^NS^p>0.2. In no case were the median pEGFR-IR scores in the Groups IIa and IIb significantly different from each other (p>0.4, Mann-Whitney U-test).

In order to visualise the importance of these findings, survival curves were constructed for the 235 cases scored for both pEGFR-IR and CB_1_IR. The data was divided into four subsets on the basis of the pEGFR-IR and CB_1_IR scores. The Groups are termed Ia (n = 101), Ib (n = 7), IIa (n = 98) and IIb (n = 29), where I and II refer to the pEGFR-IR (<3.2 and >3.2, respectively) and a and b refer to the CB_1_IR scores. Thus, for example, Group Ia consists of cases where both scores are below their respective Youden cut-offs whilst Group IIb represents the other extreme, where both scores are above the respective Youden cut-offs. The very low incidence of cases in Group Ib (low pEGFR-IR, high CB_1_IR) means that the survival curves for this group are much less robust than for the other groups. Nonetheless, a clear pattern emerged, consistent with the COX regression analyses, where the disease-specific survival was best for the Group Ia cases and poorest for the Group IIb (and possibly also Group Ib) cases (Fig. 3A). The 15 year disease-specific survival for Groups Ia, IIa and IIb were 85±5%, 54±7% and 7±6%, respectively. The corresponding value for Group Ib was 22±19%, the large s.e.m. value reflecting the small sample size. The pattern whereby a high CB_1_IR augmented the effect of a high pEGFR-IR (i.e. Groups IIa vs IIb) was also seen when the data was restricted to subsets of cases with tumour stage T2 (Fig. 3B), Gleason scores 6–7 (Fig. 3C), and 8–10 (Fig. 3D). Comparisons for other subgroups are limited either by too few events (Gleason scores 4–5, tumour stage T1a–T1b) or small group sizes (Tumour stage T3 and T4) and are in consequence not shown here.

### A high CB_1_IR augments the effect of a high pEGFR upon disease severity at diagnosis

**Figure 4:**
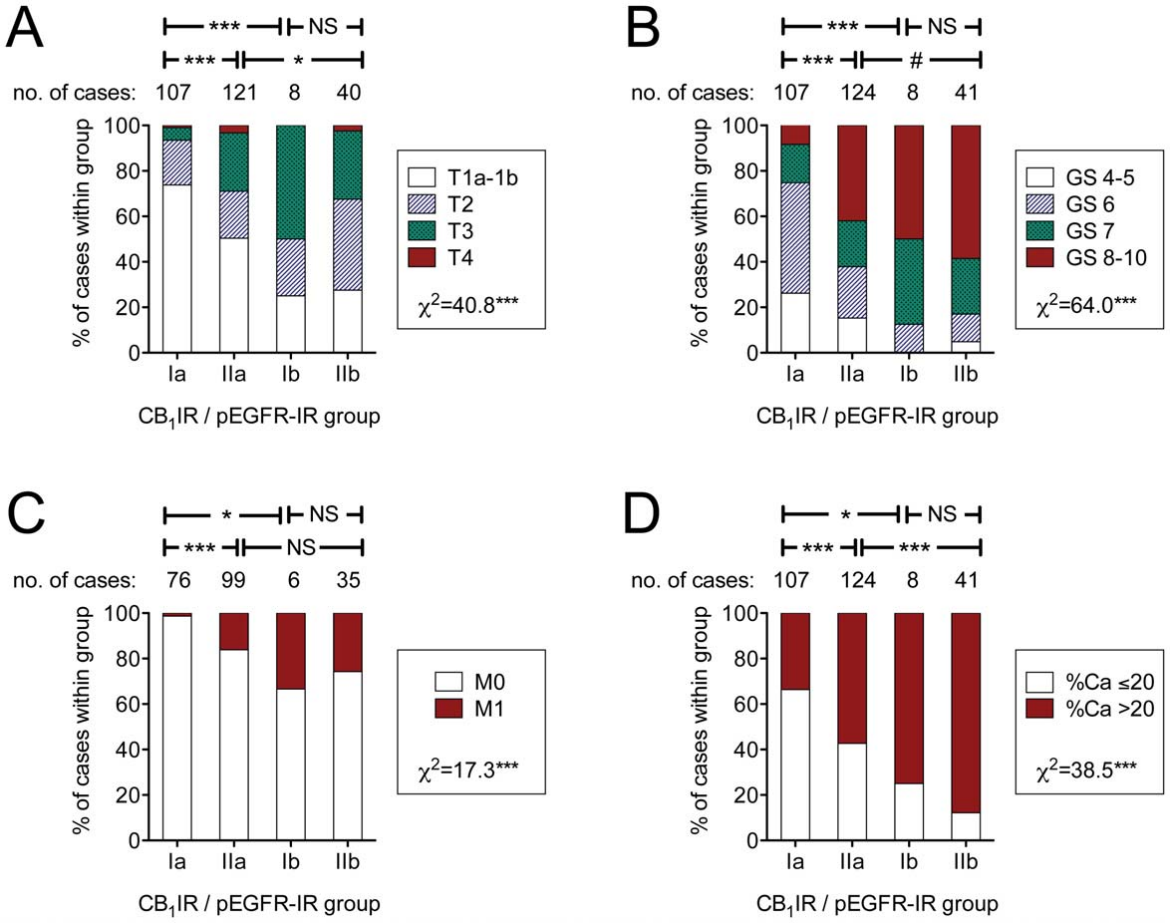
Effects of the tumour CB_1_IR and pEGFR scores upon the severity of the disease at diagnosis. Shown are: A, tumour stage (T); B, Gleason scores (GS); C, absence (M0) or presence (M1) of metastases at diagnosis; and D, the % of the specimen that was contained tumour (%Ca). For definition of the CB_1_IR/pEGFR-IR groups, see Legend to Fig. 3. The χ^2^ statistic for the total data set is given in each panel under the explanatory legends. The symbols between the capped lines above the bars indicate significance levels for the individual comparisons shown. ***p<0.001, #p<0.1, NS, p>0.2, χ^2^ test or Fisher's exact test (when comparisons were for a 2×2 matrix).

In view of the finding that the CB_1_IR provided additional prognostic information to that of pEGFR, the pattern of disease severity at diagnosis was investigated for all 280 patients scored for both parameters. Again, the number of cases in Group Ib was low (n = 8). Nonetheless, for the four markers of disease severity investigated (Gleason score, tumour stage, incidence of metastases at diagnosis and the percent of the specimen that contained tumour, there was a clear influence of the CB_1_IR/pEGFR Group upon the observed pattern, with the Group IIb cases having the most severe pattern (Fig. 4). For two of the four measures, the Group IIb cases had a more severe pattern than the Group IIa cases, suggesting that the deleterious influence of a high pEGFR expression in the tumours is further augmented by a high CB_1_ expression.

## Discussion

In the present study, we have reanalysed previously published data [16], [18] to determine how the expression of prostate tumour epithelial CB_1_ receptors affects disease severity and outcome in patients with different tumour expression levels of pEGFR. In this discussion, three questions are addressed:

### 1. What was known prior to the analyses and what is novel?

We had previously reported that both CB_1_IR and pEGFR-IR are associated with disease severity at diagnosis and with disease-specific survival [16], [18] and that the two measures are correlated [19]. This correlation might have meant that the two markers act simply as alternative prognostic markers and that the utility of one in a diagnostic test would not be improved by the addition of the second marker. In fact, the present study shows that they provide additive diagnostic information, which may be useful indeed.

### 2. What are the implications of the study for disease mechanisms?

The simplest way to consider the implications of the present study is to consider cancer cells that are susceptible to the deleterious effects of CB receptor activation and those that are not. In susceptible cell lines from several different cancer types, activation of CB receptors leads to a variety of different cellular events, including the sustained production of ceramide, reduced expression of vascular endothelial growth factor and matrix metalloprotease-2, and the sustained activation of extracellular signal-related kinase 1/2, resulting in apoptosis, inhibition of tumour adhesion, migration and angiogenesis [2], [23], [24]. *In vitro* studies undertaken in cell lines together with data from tissue microarrays are consistent with the proposal that a local endocannabinoid tonus controls the invasivity of prostate cancer cells [10]–[12], [19] (see introduction). In this respect, the endocannabinoid system can be considered as a “damage limiter” not only in prostate cancer [10]–[12], but in other potentially harmful situations, such as following tissue damage [25]. This “damage limitation” may also occur in some other solid tumours, given than in hepatocellular cancer, a low CB_1_ receptor expression impacts negatively upon survival [26], and that in colorectal cancer, a loss of CB_1_ receptors due to hypermethylation of the CB_1_ receptor promotor region has been reported [27]. However, such “damage-limitation” can be negated by overexpression of other pathways promoting cell proliferation and survival. The EGFR receptor is coupled to a number of intracellular signalling systems, such as the Ras/Raf/MAPK and PI3K/Akt pathways, which induce cell proliferation, migration and resistance to apoptosis [13], [14]. In rat C6 glioma cells, the level of expression of the EGFR ligand amphiregulin is a factor determining the degree of resistance of the cells to the deleterious effects of cannabinoids [28]. Extrapolating this finding to the prostate (with all the appropriate caveats concerning different cancer cell types and the large step between cultured cells and tumour tissue), it can be argued that overexpression of EGFR ligands, the EGFR receptor itself, and/or the level of EGFR activation would work against the local protective endocannabinoid tone. Certainly, this would be consistent both with the *in vitro* study showing that anandamide down-regulates EGFR [5], and may contribute to some extent to the more severe form of the disease seen at diagnosis for patients with a high pEGFR-IR (Fig. 4, comparison between Groups Ia and IIa). With respect to disease-specific survival, there is clearly an effect of the pEGFR for the entire data set (Table 1, Fig. 3A), although this is not seen for the Gleason group 6–7 and the tumour stage 2 cases.

The observation in the present study that a high, rather than a low, CB_1_IR compounds the effect of pEGFR upon disease severity and outcome is at first sight rather difficult to explain, since a high CB_1_ receptor expression would be expected to be protective, rather than damaging. However, an attractive explanation can be formulated on the basis of recent data from astrocytoma cells [29], where the responsiveness to cannabinoids was found to be dependent upon the expression level of CB receptors. These authors showed at low levels of CB receptor expression, the predominant signalling pathway was via Erk1/2, and cannabinoids produced apoptosis, whilst at high levels of expression, a second signal transduction pathway via Akt (a survival pathway) became predominant, and the ability of cannabinoids to produce apoptosis was lost, unless Akt signalling was blocked concomitantly [29]. Taken together, these data would suggest that the ability of endocannabinoids to act as a local regulator limiting the spread of cancer cells would be replaced by a pro-survival effect of these local mediators at high rates of receptor expression. A mitogenic effect of cannabinoids in LNCaP cells has been reported [9] and it is possible that this may also be related to the level of expression of CB receptors in the cells under the conditions used. This hypothesis is admittedly based on work with cultured cells, but would explain why a high expression of CB_1_ receptors is associated with a poor disease-specific survival in both prostate cancer [18] and pancreatic cancer [30]. In such cases, a high CB_1_ receptor expression would compound the deleterious (and non-CB_1_ receptor-related) effects produced by a high EGFR activity. A high expression of phosphorylated Akt (pAkt-1) is associated with a poorer recurrence-free survival in prostate cancer [31], and it would clearly be of interest to investigate whether the expression of CB_1_ receptors is correlated with pAkt-1 in prostate tumour tissue obtained at diagnosis. Additionally, it would be of interest to determine in cultured cells expressing high levels of CB_1_ receptors whether activation of these receptors results in an increased phosphorylation of EGFR, since this would provide an explanation for the low incidence of cases with a high CB_1_IR/low pEGFR-IR in the present study.

### 3. Do CB_1_IR and pEGFR-IR have diagnostic potential?

We have previously argued that the pEGFR score may be a useful measure to aid treatment decisions for patients with Gleason scores 6 or 7 [16]. The present study would condition that conclusion somewhat, suggesting that the CB_1_IR is of considerable importance. This is particularly true for the entire data set, where the 15 year disease-specific survival for patients with low pEGFR-IR and CB_1_IR scores (Group Ia) is very favourable (85±5%). In contrast, patients with high expressions of the two parameters (Group IIb) had a very poor 15 year disease-specific survival (7±6%), with patients with a high pEGFR-IR and a low CB_1_IR (Group IIa) being intermediate (54±7%). This, together with the fact that the two measures provide additional prognostic information not only to each other but importantly to that given by the Gleason scores and the tumour stage (Table 1) raises the possibility that they may have useful diagnostic value in aiding treatment decisions, the proviso, of course, being that other researchers can duplicate our findings in different patient samples and with different (but equally well validated) antibodies. However, a practical hinder to their use is that the scores are a composite of immunoreactive intensity and distribution, which may be cumbersome in a clinical setting. What needs to be determined is the extent to which the scores can be simplified without losing their diagnostic power. We have started to investigate this with respect to the CB_1_IR, and found that reanalysis of selected cores for each case using predominant intensity as a measure instead of the composite score does retain some of its diagnostic power (association with disease severity and outcome, as well as its ability to provide added diagnostic information to that provided by the tumour stage), whereas its ability to provide added diagnostic information to that provided by the Gleason score is lost (C.J. Fowler, unpublished findings). However, given the clear influence of pEGFR-IR and CB_1_IR upon disease severity and outcome, studies optimizing these markers for clinical use are clearly warranted.

## Supporting Information

Figure S1 **CB_1_IR as a prognostic factor.** A useful way to assess the prognostic value of a biomarker is to select a cut-off value from a data subset and then validate it using a separate data subset (see [16] for an example with pEGFR). Here, the 419 original cases were assigned a random number (using different random sets for CB_1_IR and pEGFR-IR) and the untreated patients in the random number set 1-279 and 280-419 were used as the test and validation sets, respectively. Panel A shows the ROC curve (using a 15 year limit) for CB_1_IR in the test set, from which the optimal cutoff (Youden index, shown as an arrow in the figure) at >2.3, i.e. the same as for the complete data set (see Results), was chosen. Panels B and C show Kaplan-Meier plots for the test set and validation set, respectively. †_p_ refers to the number of cases who died as a result of the prostate cancer. The χ^2^ values shown in the panels are from the log rank (Cox-Mantel) test. Thus, the survival curves for both the test and the validation sets using these cut-offs confirmed the prognostic value of CB_1_IR.
(TIF)
