# Supplementary figures and images for: Tumour Cannabinoid CB1 Receptor and Phosphorylated Epidermal Growth Factor Receptor Expression Are Additive Prognostic Markers for Prostate Cancer
Source: PLoS One. 2010 Dec 23;5(12):e15205. doi: 10.1371/journal.pone.0015205 (PMC3009725; doi:10.1371/journal.pone.0015205)

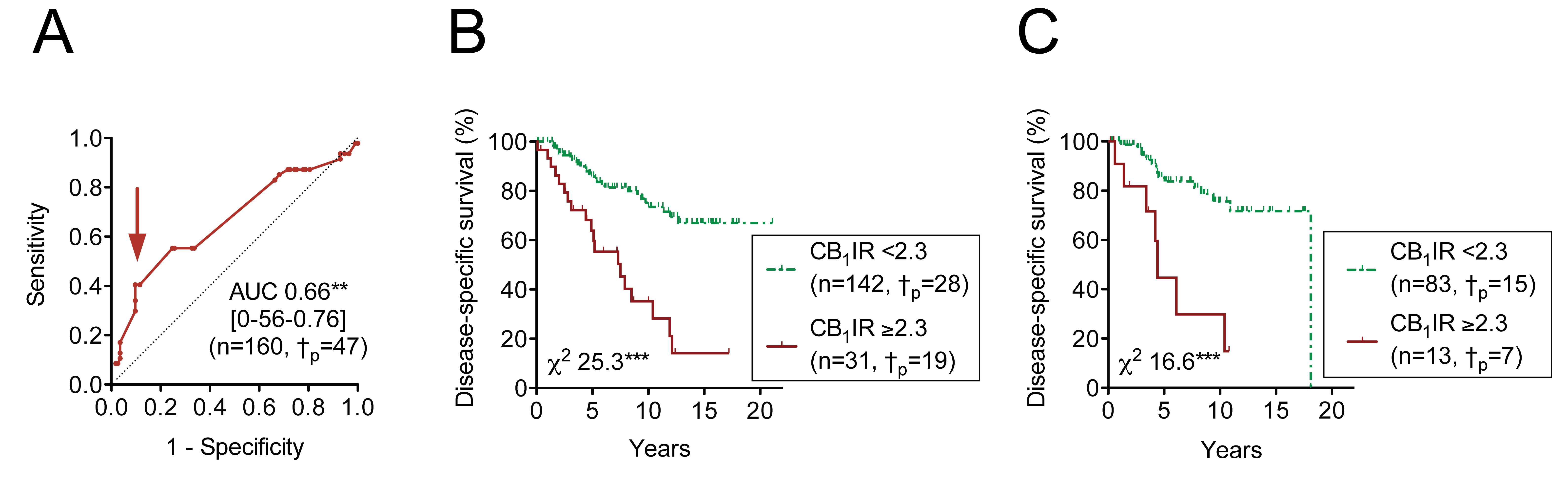

Supplement: Figure S1 — CB1IR as a prognostic factor. A useful way to assess the prognostic value of a biomarker is to select a cut-off value from a data subset and then validate it using a separate data subset (see [16] for an example with pEGFR). Here, the 419 original cases were assigned a random number (using different random sets for CB1IR and pEGFR-IR) and the untreated patients in the random number set 1-279 and 280-419 were used as the test and validation sets, respectively. Panel A shows the ROC curve (using a 15 year limit) for CB1IR in the test set, from which the optimal cutoff (Youden index, shown as an arrow in the figure) at >2.3, i.e. the same as for the complete data set (see Results), was chosen. Panels B and C show Kaplan-Meier plots for the test set and validation set, respectively. †p refers to the number of cases who died as a result of the prostate cancer. The χ2 values shown in the panels are from the log rank (Cox-Mantel) test. Thus, the survival curves for both the test and the validation sets using these cut-offs confirmed the prognostic value of CB1IR. (TIF) [file pone.0015205.s001.tif]
